# Use of snus, its association with smoking and alcohol consumption, and related attitudes among adolescents: the Finnish National School Health Promotion Study

**DOI:** 10.1186/s12971-015-0058-3

**Published:** 2015-10-24

**Authors:** Battsetseg Tseveenjav, Paula Pesonen, Jorma I. Virtanen

**Affiliations:** Dental Health Care, Division of Health and Substance Abuse Services, Department of Social Services and Health Care, City of Helsinki, FI-00099 Helsinki, Finland; Research Unit of Oral Health Sciences, Faculty of Medicine, University of Oulu, FI-90014 Oulu, Finland; Oulu University Hospital, FI-90029 Oulu, Finland; Medical Research Center (MRC Oulu), Oulu, Finland

**Keywords:** Adolescents, Alcohol consumption, Non-conventional tobacco product, Smokeless tobacco, Parental education, Smoking, Use of snus

## Abstract

**Background:**

The relationship between the use of snus and lifestyle-related habits – especially in adolescence, when these behaviours begin and become established – is not widely studied. Our aim was to analyse associations between snus use and habits of and attitudes towards smoking and alcohol consumption among Finnish adolescents.

**Methods:**

The study is a part of the National School Health Promotion Study in Finland. The study population consisted of a representative sample of Finnish adolescents (*n* = 183 226). A questionnaire enquired about pupils’ use of snus, habits of and attitudes towards smoking and alcohol consumption, as well as their (age, gender, school type) and their parents’ (education and smoking) background factors. Chi-square tests and logistic regression models served in the statistical analyses.

**Results:**

Of the adolescents, 18 % had used snus (2 % daily, 16 % experimented) while 82 % never had. Snus use was more common among boys than girls (*p* < 0.05). Concerning smoking, 19 % were daily and 15 % occasional smokers. Regarding alcohol, 11 % consumed it weekly and 57 % monthly or less frequently. More than two thirds of the adolescents held positive attitudes towards smoking (71 %), and alcohol (67 %). Male gender (OR = 9.9; 95 % CI 9.4–10.4), current (OR = 32.8; 95 % CI 26.1–41.1) or former (OR = 10.1; 95 % CI 8.0–12.9) smoking, weekly consumption of alcohol (OR = 27.4; 95 % CI 21.0–35.8), positive attitude towards smoking (OR = 1.4; 95 % CI 1.3–1.6), and higher parental education (OR = 1.4; 95 % CI 1.3–1.4) associated significantly with adolescents’ current snus use, whereas parental smoking did not.

**Conclusion:**

Current snus use among adolescents may signal an accumulation of other lifestyle-related risky behaviours such as current or past smoking and alcohol consumption as well as a positive attitude towards smoking. In addition to these possible co-existing health-related risk factors, health promotion activities should take into account gender and school differences in order to target preventive messages to youth more effectively.

## Background

Although the prevalence of cigarette smoking among adults has declined in recent years in many industrialised countries in Northern and Western Europe [1], the use of a diversity of non-conventional tobacco products has become commonplace and increased in many countries such as Sweden [2], Norway [3, 4], and Finland [5, 6] in recent decades.

The current literature seems to reveal some controversy about whether smokeless tobacco use is a gateway to cigarette smoking; some studies conclude that smokeless tobacco use does not associate with subsequent smoking [7–9], whereas others suggest that it does [10–13].

There is, however, scientific consensus that the cancer risk for non-conventional tobacco use is lower than for smokers, but higher than for non-smokers [14]. In addition, to nicotine addiction [15], for example, snus use causes easily detectable oral soft tissue lesions [16], and smokeless tobacco generally raises the risk for several diseases [14, 17–19].

In Finland, snus use among youth is increasing despite a European Union (EU) ban on the sale of snus since Finland joined the EU in 1995 [5]. The most commonly used type is Swedish snus which is used either alone or along with cigarette smoking (dual use) [15]. Such use is more common among boys than girls, with daily use ranging from 12 to 18 % among boys [16] and 2 % among girls [5].

Social, lifestyle and health profiles of exclusive snus users are reportedly less favourable than those of non-users, but more advantageous than those of exclusive smokers among adults [20]. Among adolescent boys in Sweden, the snus use indicates drug- and risk-seeking lifestyle [21]. Finnish young men show a clear association between the use of snus and team sports [22]. Daily smoking among adolescents is known to negatively associate with socio-educational backgrounds, but little is known about such associations for snus. Snus use may indicate low educational ambitions and less affluent self-reported family economy [23]; however it seems to deviate from how smoking is distributed across social strata [3].

Most lifestyle-related behaviours, especially the use of snus and smoking habits, begin and become established in adolescence [24, 25]. Our general aim was to investigate the relationship between snus use and other lifestyle-related behaviours and attitudes among adolescents. A specific aim was to associate Finnish adolescents’ snus use with their habits of and attitudes towards cigarette smoking and alcohol consumption, taking into account their parental background such as education and smoking habits. Our working hypothesis was that snus use among adolescents is related to their smoking and alcohol consumption as well as to their attitudes towards these habits, and hence the accumulation of lifestyle-related risky behaviours.

## Methods

### Study subjects

The School Health Promotion Study has taken place systematically since the year 2000 in an effort to monitor the health and wellbeing of Finnish 14- to 20-year-old adolescents and to strengthen the planning and evaluation of oral health promotion activities at the school, municipal and national levels [26]. The nationwide study takes place every second year and surveys adolescents’ living conditions, school experiences, and health as well as their health-related behaviours. The present cross-sectional study is a part of the National School Health Promotion Study and was conducted in 2010–2011. The Ethics Committee of the National Institute for Health and Welfare, Finland, approved the study.

The study population consisted of a representative sample of Finnish adolescents from three different school types: 8^th^ and 9^th^ grade pupils from comprehensive school and 1^st^ and 2^nd^ year students from upper secondary and vocational schools. Altogether, 192 414 adolescents anonymously replied to the questionnaire. The study reached 80 % of comprehensive and 73 % of upper secondary school adolescents (it was not possible to calculate the percentage for vocational school). For this study, the selection of adolescents between 13 and 19 years of age yielded a sample of 183 226.

### Questionnaire

An anonymous, classroom-administered questionnaire served to gather the data. The survey was confidential, and participation was voluntary. The most recent version of questionnaire in English is available on the internet pages of the survey [26]. (http://www.thl.fi/attachments/kouluterveyskysely/SHP_questionnaire_2013.pdf).

The questionnaire queried adolescents about their background information, snus use, smoking habits and alcohol consumption as well as their parents’ smoking habits using a Likert scale.

The question “Do you use snus?” asked adolescents to rate their snus use on a four-point Likert scale: not at all, once, sometimes, or daily.

The question “Which of following alternatives best describes your current smoking habits?” enquired about smoking, with the following answer alternatives: daily, once or more weekly, less than once a week, not currently, or not at all.

The question “How often do you use alcohol, such a half-bottle of beer or more?” with the following answer alternatives: once a week or more, twice monthly, once a month, less than once a month, or not at all.

The question “Do you generally acquiesce if someone sometimes smokes?” enquired about adolescents’ attitudes towards smoking, with three response categories: 1) Yes, I do; 2) No, I don’t; and 3) I don’t know. The question “Do you generally acquiesce if someone drinks a couple of shots of alcohol weekly?” enquired about adolescents’ attitudes towards alcohol use, with the same response categories as in the previous question.

Adolescents’ parental education and smoking habits was enquired separately for each parent, originally across four categories: primary school, secondary (upper or vocational training), secondary school and vocational degree, and university or polytechnics.

### Variable regrouping

For further analyses, we regrouped the original scales as follows: scales for the question on snus use was converted into three descriptive categories: current snus users (daily and occasional users), tried once, and not at all. In cross-tabulations and logistic models, snus use served as a binary variable (current snus use vs. never tried or once). Those who tried snus, but did not use it daily, fell under the category of snus experimenters. We reclassified smoking frequency into three categories: current smokers (those who smoked daily, once or more weekly, or less than once weekly), not currently smoking, and not at all. We regrouped alcohol consumption into four categories: weekly, monthly, less than monthly, and not at all. We also dichotomised attitude variables for smoking and alcohol, as follows: positive attitude (“Yes, I do”) vs. else (“No, I don’t” or “I don’t know”). Further, we created and coded a parental smoking variable as “yes” if one of the parents was a current smoker. For parental education level, we created and coded a new variable as follows: “higher”, if at least one of the parents had completed a vocational, university or polytechnics degree, vs. else.

### Background information

The adolescents’ age, gender and school type served as background variables. Their mean age by school type was 15.4 (SD = 0.6) for CS (*n* = 99 597), 17.3 (SD = 0.6) for USS (*n* = 46 644) and 17.4 (SD = 0.7) for VS (*n* = 36 985) adolescents; 50 % were boys.

### Statistical analyses

The statistical significance of the differences in frequencies was determined with the chi-square test at a significance level of *p* < 0.05. Because of gender differences in snus use, all analyses were run separately by gender. Multivariate logistic regression models were served to associate snus use with an adolescent’s gender, school type, smoking and alcohol consumption as well as attitudes towards these habits, in addition to parental education and parental smoking. The outcome of the dependent variable was “current use of snus”. All explanatory variables served as categorical variables. The reference groups of the explanatory variables were “girls” for gender, “comprehensive school” for school type, “not at all” for both smoking and alcohol use, and “else” for attitude variables. Concerning parent-related variables, the reference groups were “current smoking” for parental smoking and “primary or secondary school” for parental education. Models were adjusted according to the adolescents’ age and all statistically significant two-way interaction terms.

## Results

Table 1 shows the adolescents’ snus use, smoking and alcohol consumption by school type. Snus use was more frequent among boys than girls (*p* < 0.001). The prevalence of “tried snus once” among the girls (13 %) was as high as among the boys (12 %) in vocational schools. Of all the adolescents, 19 % smoked daily, and 15 %, occasionally (5 % weekly, 10 % less than weekly). Of the adolescents, 11 % consumed alcohol once or more weekly, 23 % twice monthly, 13 % once monthly and 21 % less than once monthly. Almost one third consume no alcohol at all.

**Table 1:** Adolescent- and parent-related variables by off-springs’ school type (*N* = 183,226)

	Comprehensive		Upp. secondary		Vocational		All		
	(*n* = 99,597)		(*n* = 46,644)		(*n* = 36,985)				
	Boys %	Girls %	Boys %	Girls %	Boys %	Girls %	Boys %	Girls %	All %
Snus use									
Daily	3	<1	6	<1	5	<1	4	<1	2
Sometimes	12	2	14	3	21	4	12	2	7
Tried once	10	7	10	9	12	13	10	8	9
Not at all	75	91	70	88	62	83	74	90	82
*p*-value	<0.001		<0.001		<0.001		<0.001		
Smoking									
Daily	16	13	10	11	40	40	20	17	19
Weekly	4	5	5	5	5	5	5	5	5
Less than weekly	7	10	12	15	7	9	9	11	10
Not currently	15	13	16	15	15	16	15	14	15
Not at all	58	59	57	54	33	30	51	52	51
*p*-value	<0.001		<0.001		<0.001		<0.001		
Alcohol use									
Weekly or more	8	6	14	9	25	18	13	9	11
Twice monthly	16	17	29	30	31	33	22	23	23
Once monthly	10	12	14	18	13	15	12	14	13
Less than monthly	22	23	18	21	15	19	20	22	21
Not at all	44	42	25	22	16	15	33	32	32
*p*-value	<0.001		<0.001		<0.001		<0.001		
Attitude towards smoking									
Yes, I accept smoking	65	65	74	71	82	84	71	70	71
Else^a^	35	35	26	29	18	16	29	30	29
*p*-value	0.027		<0.001		<0.001		<0.001		
Attitude towards alcohol use									
Yes, I accept the use	70	56	80	63	78	65	74	60	67
Else^b^	30	44	20	37	22	35	26	40	33
*p*-value	<0.001		<0.001		<0.001		<0.001		
Parental smoking									
Yes^a^	35	37	24	28	43	48	35	36	35
No	65	63	76	72	57	52	65	64	65
*p*-value	<0.001		<0.001		<0.001		<0.001		
Parental education^c^									
Primary or secondary	59	63	46	52	74	80	60	63	61
Higher education	41	37	54	48	26	20	40	37	39
*p*-value	<0.001		<0.001		<0.001		<0.001		

Adolescent-related variables: adolescents’ snus use, smoking and alcohol consumption as well as attitudes towards smoking and alcohol; parent-related variables: parental smoking and education; School type: *CS* comprehensive school, *USS* upper secondary school, *VS* vocational school; Statistical evaluation was done by chi-square test

^a^At least one of the parents smoked

^b^Else: answer included options: I don’t accept or I don’t know

^c^The highest educational level at least one of the parents completed (Primary or secondary level included CS, USS, VS; Higher level included: vocational degree or university or polytechnics)

Concerning attitudes towards smoking and alcohol consumption, more than two thirds of the adolescents accepted that people smoke occasionally (71 %) and that people drink a couple of shots per week (67 %) (Table 1). Their attitudes to both smoking and alcohol differed by gender and school type. More than one third (35 %) of the adolescents had at least one smoker parent. Adolescents in vocational school more often had at least one smoking parent. Almost two thirds of the adolescents in this study had at least one parent with a higher educational level.

Among both boys and girls, comprehensive school adolescents were more likely to be “not at all” snus users, whereas their vocational school counterparts were more likely to be “current” snus users (*p* < 0.001).

Adolescents’ “current snus use” related strongly to their smoking habits and attitudes towards smoking (Table 2). Current snus users tended to be current smokers and to have positive attitudes towards smoking, a finding that proved consistent across all school types and both genders.

**Table 2:** Adolescents' snus use by their smoking and attitudes towards smoking (*N* = 183,226)

	Smoking (%)				Attitudes towards smoking (%)		
	Current smoker	Not currently	Not at all	*p*-value	Yes, I accept the use	Else	*p*-value
Comprehensive (*n* = 99,597)							
Boys (all)	27	15	58		65	35	
Current snus user	71	18	11	<0.001	88	12	<0.001
Never tried or once	20	14	66		61	39	
Girls (all)	28	13	59		65	35	
Current snus user	88	9	3	<0.001	96	4	<0.001
Never tried or once	27	13	60		64	36	
Upper secondary (*n* = 46,644)							
Boys (all)	28	16	56		74	26	
Current snus user	65	22	13	<0.001	90	10	<0.001
Never tried or once	18	15	67		70	30	
Girls (all)	31	15	54		71	29	
Current snus user	75	17	8	<0.001	92	8	<0.001
Never tried or once	30	15	55		71	29	
Vocational school (*n* = 36,985)							
Boys (all)	51	15	34		82	18	
Current snus user	79	14	7	<0.001	92	8	<0.001
Never tried or once	42	15	43		79	21	
Girls (all)	54	16	30		84	16	
Current snus user	89	9	2	<0.001	96	4	<0.001
Never tried or once	53	16	31		84	16	

Comparisons in each school type, by gender; statistical evaluation was done by chi-square test

Regarding the adolescents’ snus use according their alcohol consumption and attitudes towards alcohol use, current snus users were more likely to consume alcohol weekly or monthly and to hold positive attitudes towards alcohol use (Table 3), a finding that proved consistent across all school types and both genders.

**Table 3:** Adolescents’ snus use by their alcohol consumption and attitudes towards alcohol (*N* = 183,226)

	Alcohol consumption (%)					Attitudes towards alcohol use (%)		
	Weekly	Monthly	Less than monthly	Not at all	*p*-value	Yes, I accept the use	Else	*p*-value
Comprehensive (*n* = 99,597)								
Boys (all)	8	26	22	44		70	30	
Current snus user	24	50	18	8	<0.001	76	24	<0.001
Never tried or once	5	22	23	50		69	31	
Girls (all)	6	29	23	42		56	44	
Current snus user	38	50	10	2	<0.001	73	27	<0.001
Never tried or once	5	29	23	43		56	44	
Upper secondary (*n* = 46,644)								
Boys (all)	14	43	18	25		80	20	
Current snus user	31	60	7	2	<0.001	82	18	0.003
Never tried or once	10	39	21	30		80	20	
Girls (all)	9	48	21	22		63	37	
Current snus user	33	59	7	1	<0.001	72	28	<0.001
Never tried or once	8	47	22	23		62	38	
Vocational school (*n* = 36,985)								
Boys (all)	25	43	15	16		78	22	
Current snus user	41	48	7	4	<0.001	82	18	<0.001
Never tried or once	20	42	18	20		77	23	
Girls (all)	18	48	19	15		65	35	
Current snus user	40	47	9	4	<0.001	76	24	<0.001
Never tried or once	17	49	19	15		64	36	

Comparisons in each school type and by gender; Statistical evaluation was done by chi-square test

Current snus-using adolescents were more likely to have at least one smoking parent, a finding that was consistent across all school types and both genders (Table 4). Concerning parental education, current snus-using boys were more likely to have at least one parent who achieved a higher educational level.

**Table 4:** Adolescents’ snus use by parental smoking and education (*N* = 183,226)

	Parental smoking^a^ (%)			Parental education^b^ (%)		
	yes^b^	no	*p*-value	Primary or secondary school	Higher educational level	*p*-value
Comprehensive (CS) (*n* = 99,597)						
Boys (all)	35	65		60	40	
Current snus user	44	56	<0.001	57	43	<0.001
Never tried or once	34	66		60	40	
Girls (all)	37	63		63	37	
Current snus user	55	45	<0.001	62	38	0.606
Never tried or once	36	64		63	37	
Upper secondary (USS) (*n* = 46,644)						
Boys (all)	24	76		46	54	
Current snus user	27	73	<0.001	39	61	<0.001
Never tried or once	24	76		47	53	
Girls (all)	28	72		52	48	
Current snus user	34	66	<0.001	50	50	0.148
Never tried or once	28	72		52	48	
Vocational school (VS) (*n* = 36,985)						
Boys (all)	43	57		75	25	
Current snus user	48	52	<0.001	69	31	<0.001
Never tried or once	41	59		76	24	
Girls (all)	48	52	<0.001	80	20	
Current snus user	57	43		77	23	0.016
Never tried or once	48	52		80	20	

Comparisons in each school type, by gender of adolescents

Statistical evaluation was done by chi-square test

^a^At least one of the parents smoked

^b^The highest educational level at least one of the parents achieved

(Primary or secondary level included CS, USS, VS; Higher level included: vocational degree or university or polytechnics)

The multivariate logistic regression analyses, when adjusted for age and interaction terms, showed that current snus use was more common among boys (OR = 9.9) than among girls, and among vocational school adolescents (OR = 1.4) than among their comprehensive school counterparts (Table 5). Smoking frequency and alcohol consumption strongly associated with current snus use. Currently smoking adolescents were more likely to be current snus users than were those who did not smoke at all. Alcohol use associated with current snus use; adolescents who consumed alcohol were more likely to be current snus users than were those who consumed no alcohol at all. A positive attitude towards smoking associated with current snus use, but a positive attitude towards alcohol did not. These associations remained statistically significant even when logistic models were applied separately by school type. Of parent-related factors, higher parental education compared to a primary or secondary education level associated with current snus use among their offspring, an association which remained statistically significant across all school types.

**Table 5:** Adolescents’ current use of snus explained by adolescent- and parent-related factors

	^a^All	^b^Comprehensive	^b^Upper secondary	^b^Vocational
	(*N* = 183,226)	(*N* = 99,597)	(*N* = 46,644)	(*N* = 36,985)
	OR (95 % CI)	OR (95 % CI)	OR (95 % CI)	OR (95 % CI)
Gender				
boys vs. girls	9.9 (9.4–10.4)	10.3 (9.6–11.0)	11.1 (10.1–12.1)	8.4 (7.7–9.2)
School type				
USS vs. CS	0.8 (0.6–1.0)	-	-	-
VS vs. CS	1.4 (1.1–1.8)			
Smoking				
current smoker vs. not at all	32.8 (26.1–41.1)	29.2 (21.9–38.8)	50.7 (27.9–91.9)	39.3 (23.0–67.3)
not currently vs. not at all	10.1 (8.0–12.9)	8.9 (6.5–11.7)	13.7 (7.1–26.1)	16.8 (9.5–29.9)
Alcohol consumption				
weekly or more vs. not at all	27.4 (21.0–35.8)	21.5 (14.7–31.5)	21.3 (11.8–38.6)	16.4 (9.4–28.6)
monthly vs. not at all	13.5 (11.0–16.6)	12.3 (9.6–15.7)	14.0 (8.6–23.0)	9.9 (6.1–16.3)
less than monthly vs. not at all	5.9 (4.7–7.3)	5.4 (4.2–6.9)	5.1 (3.0–8.8)	4.5 (2.6–7.9)
Attitudes towards smoking				
positive vs. else	1.4 (1.3–1.6)	1.4 (1.2–1.7)	1.4 (1.1–1.7)	1.4 (1.03–1.7)
Attitudes towards alcohol use				
positive vs. else	0.6 (0.5–0.7)	0.6 (0.5–0.7)	0.3 (0.2–0.6)	0.7 (0.5–1.0)
Parental smoking^c^				
current smoker vs. else	1.0 (0.98–1.1)	1.02 (0.96–1.1)	0.99 (0.91–1.1)	1.1 (0.98–1.1)
Parental education^d^				
higher vs. primary or secondary	1.4 (1.3–1.4)	1.4 (1.3–1.5)	1.3 (1.2–1.4)	1.4 (1.3–1.5)

*OR* Adjusted odds ratios with 95 %, *CI* confidence intervals, School type: *CS* comprehensive school, *US* upper secondary school, *VS* vocational school

^a^Logistic model adjusted by age and interaction terms; Interaction terms used for the models: school type*smoking; school type*alcohol consumption; smoking*alcohol consumption; smoking* attitude towards smoking; smoking*attitude towards alcohol consumption; alcohol consumption* attitude towards alcohol consumption

^b^Logistic models adjusted by age and interaction terms; Interaction terms used for the models: smoking*alcohol consumption; smoking* attitude towards smoking; smoking*attitude towards alcohol consumption; alcohol consumption* attitude towards alcohol consumption

^c^At least one of the parents smoked

^d^The highest educational level at least one of the parents achieved

(Primary or secondary level included CS, USS, VS; Higher level included vocational degree or university or polytechnics)

## Discussion

Our study showed that current snus use associated independently with cigarette smoking and alcohol consumption among Finnish adolescents regardless of school type. Adolescents’ current snus use also associated consistently with a positive attitude towards smoking, but not with a positive attitude towards alcohol. These associations support our working hypotheses that lifestyle-related risky behaviours accumulate among youth.

The accumulation or co-existence of these risky behaviours in our study is in line with earlier findings, suggesting that snus use also signals a drug- and risk-seeking lifestyle among adolescent boys [21] and that the health profiles of snus users are less favourable than those of non-users [20]. Possible explanations for this phenomenon could be personality traits, depressive symptoms or other mental health problems [27, 28]. If the adolescents continue their snus or substance use they may have worse health outcomes in the future.

Strong association between snus use and smoking and alcohol consumption habits among our study subjects is in agreement with findings in Swedish [29], Norwegian [4], and American [30] studies among adolescents. However, due to the cross-sectional design of this study, it is not possible to determine the chronological sequence of health-related risky behaviours (i.e., which habits precede the others). It is worth mentioning that among our study subjects, the heavier the drinking habit, the higher the likelihood of being a current snus user. This finding is in line with a Swedish finding among 15- to 16-year-old Swedish adolescents that, compared to non- or minimal drinkers, heavy drinkers of alcohol were at higher risk for snus use [29].

As in previous Finnish studies, in our study, dual use (snus use alongside cigarette smoking) was more common than exclusive use, independently of the adolescents’ gender or school type [15, 16]. Proportion of current snus users who smoked among this study adolescents was as high as among 9^th^ grade Stockholm boys [29].

Although Finland bans the sale of snus, the number of daily users and experimenters of snus indicates that youth easily obtain it. The main route seems to be from Sweden [31], which is the exception country in the EU that is allowed to sell snus. In general, many countries permit the purchase or distribution of unregulated non-conventional tobacco products with no age restrictions [6]. It is noteworthy that the prevalence of snus experimenters in our study was much higher than that of daily users, regardless of gender and school type. This suggests high youth awareness of snus and potential interest in it; however the phenomenon seems different from that of smoking. Nevertheless, because of the possibility that youth may misinterpret “less harmful” as meaning “safe”, messages promoting oral health should be clear and well-targeted. The International Agency for Research on Cancer [32], the U.S. Surgeon General [33], and the National Toxicology Program [34] all classify smokeless tobacco products as a human carcinogen.

Surprisingly, adolescents in general held very positive attitude towards smoking and alcohol consumption independently of school type. Positive attitudes towards smoking and alcohol use associated strongly with current snus use in univariate analyses. Interestingly, however, in multivariate analyses, a positive attitude towards smoking remained a significant factor for current snus use, unlike a positive attitude towards alcohol consumption.

The use of snus, smoking and alcohol consumption among the adolescents differed by gender and their school types, with the boys and vocational school youth engaged in these lifestyle-related habits more often than the girls and their comprehensive and upper secondary school counterparts, respectively. Differences in snus use, smoking and alcohol consumption across school types seem stem partly from differences in age. The differences in snus use, smoking and alcohol consumption across gender and school type found in this study are in line with the findings of earlier studies reporting on these health risk-related habits among Finnish youth [5, 16, 31, 35]. In Norway, snus use was more common among those planning vocational training [23]. Well-targeted public health initiatives should take into account gender- and school-based differences in adolescents’ habits.

The univariate analyses of our study showed that adolescents currently using snus were more likely to have at least one smoker parent which supports earlier findings among Swedish youth of an association between adolescents’ snus use and parental tobacco use [36]. However, this association disappeared in the multivariate analyses, which included all other explanatory variables and used several interaction terms; the models were also adjusted for age. The results from the multivariate models are in line with those of a Norwegian study, which reported no link between adolescents’ snus use and parental socio-economic status [3]. Because our questionnaire did not assess parental snus use, further studies are needed to identify this association between among Finnish adolescents between parental snus use and that of their offspring.

Concerning parental educational background, against our expectations, the univariate analysis revealed that boys, but not girls, who were current snus users more often had one parent with a higher level of education. Further, when we included other explanatory variables, used several interaction terms and adjusted the models for age, all the multivariate analyses revealed that adolescents’ current snus use consistently associated with higher parental education. Thus, our study suggests a different pattern from that of previous studies in which higher parental education was a protective factor, as in the use of e-cigarettes [6]. Including parental education as one of the factors revealed small differences in socio-economic background between Norwegian adolescent non-smokers and snus users. Further, parental background factors played a more important role among the smokers than among the snus users [4]. These results may indicate that snus truly deviates from the distribution of smoking across social strata or that snus use begins at a much earlier stage in the social diffusion process [3].

This national study, which included all school types, represents Finnish adolescents well. The anonymous survey was voluntary, and the high response rate in the comprehensive, upper secondary and vocational schools can be considered as strengths of this study. The results are therefore generalizable across the entire nation.

The cross-sectional design of this study limits the results to associations evident at specific point in time and precludes any conclusions regarding causality. However, since the School Health Survey takes place annually, it offers an opportunity for future analyses of times and trends.

As with all survey-type studies, when interpreting the results, researchers must bear in mind the limitations common to self-reported outcomes and their susceptibility to socially acceptable answering.

## Conclusions

In conclusion, adolescents’ current snus use may signal an accumulation of other lifestyle-related risk factors such as current or past cigarette smoking and alcohol consumption on a regular basis as well as positive attitudes towards smoking. In addition to these co-existing health-related risk factors, health promotion activities should take into account differences across genders and schools in order to more effectively target preventive messages to protect youth.

## Abbreviations

CI: Confidence Interval
OR: Odds-Ratio
